# Mol­ecular and crystal structure of (1*R*,3*R*,4*S*,7*R*)-3-bromo-7-(bromo­meth­yl)-1,7-dimethyl-3-nitro­bi­cyclo­[2.2.1]heptan-2-one

**DOI:** 10.1107/S2056989026003592

**Published:** 2026-04-14

**Authors:** Vitaliy A. Bilenko, Igor V. Komarov, Marian V. Gorichko, Svitlana V. Shishkina

**Affiliations:** a Taras Shevchenko National University of Kyiv, Volodymyrska Street 60, Kyiv 01601, Ukraine; b Enamine Ltd., Winston Churchill Street 78, Kyiv 02094, Ukraine; c Institute of Organic Chemistry, NAS of Ukraine, Akademik Kukhar Street 5, Kyiv 02094, Ukraine; Vienna University of Technology, Austria

**Keywords:** α-bromo ketone, α-nitro ketone, camphor derivative, mol­ecular structure, weak inter­molecular inter­actions, crystal structure

## Abstract

The bicyclic title compound, C_10_H_13_Br_2_NO_3_, is a camphor derivative, crystallizing in the Sohncke space group *P*2_1_2_1_2_1_, and the absolute configurations of its four chiral C atoms were unambiguously determined. The relative configuration of a bridgehead carbon atom was also confirmed by one- and two-dimensional NMR experiments. In the extended structure, weak C—H⋯O, C—H⋯Br and halogen bonds of the type Br⋯O consolidate the packing.

## Abstract 2

The absolute configurations of the four chiral centres of the title compound, a new camphor derivative, have been determined. Weak inter­molecular inter­actions consolidate the crystal packing.

## 1. Chemical context

Camphor is a naturally occurring renewable chiral compound readily available in both possible enanti­omeric forms. Different camphor derivatives have been applied in synthetic chemistry as chiral starting materials for numerous enanti­ospecific total syntheses of steroids (Clase & Money, 1992 ▸; Stevens *et al.*, 1983*a* ▸), terpenoids (Money & Wong, 1996 ▸; Jacobs *et al.*, 1990 ▸), vitamins (Stevens *et al.*, 1983*b* ▸, 1986 ▸; Stevens & Lawrence, 1985 ▸), as well as for the preparation of many other natural biologically active compounds and their analogues (Paquette *et al.*, 2000 ▸; García Martínez *et al.*, 2001 ▸). Bromo­camphors were used in the preparation of organocatalysts for asymmetric Michael additions based on functionalized bi­cyclo­[2.2.1]hexa­nes (Ričko *et al.*, 2015 ▸) or phosphine-carbonyl ligands for Ni-catalyzed ethyl­ene oligomerization (Behzadi *et al.*, 2020 ▸). New environmentally friendly camphor derivatives exhibit anti­fungal activity (Huang *et al.*, 2025 ▸) and have potential in activating human carbonic anhydrase, which is relevant to neurodegenerative disorders (Mishra & Sethi, 2025 ▸). Recently, *α*-bromo-*α*-nitro-ketones have shown promising bioactivity as human DNA methyl­transferase inhibitors with micromolar active concentrations for anti­cancer research and therapy (Calzaferri *et al.*, 2025 ▸; Serhouni *et al.*, 2025 ▸; Ceccaldi *et al.*, 2011 ▸; Pechalrieu *et al.*, 2020 ▸). These types of compounds also are emerging as potential anti­malarial drugs (Reyser *et al.*, 2023 ▸). In the context of expanding the specific chemical behavior and biological activity of *α*-bromo-*α*-nitro-ketones, the crystal structure of the title compound was determined.
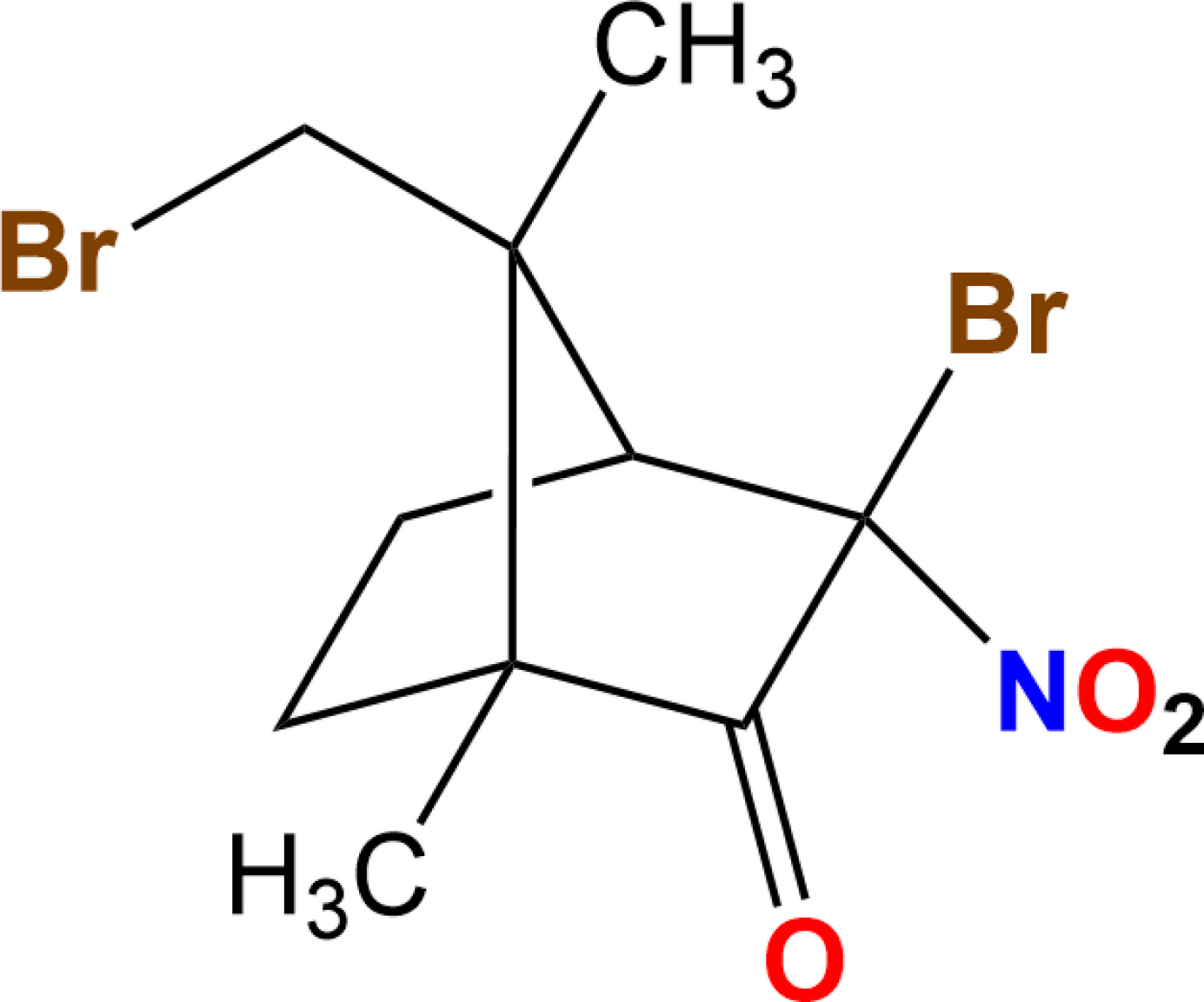


## 2. Structural commentary

**Figure 1:**
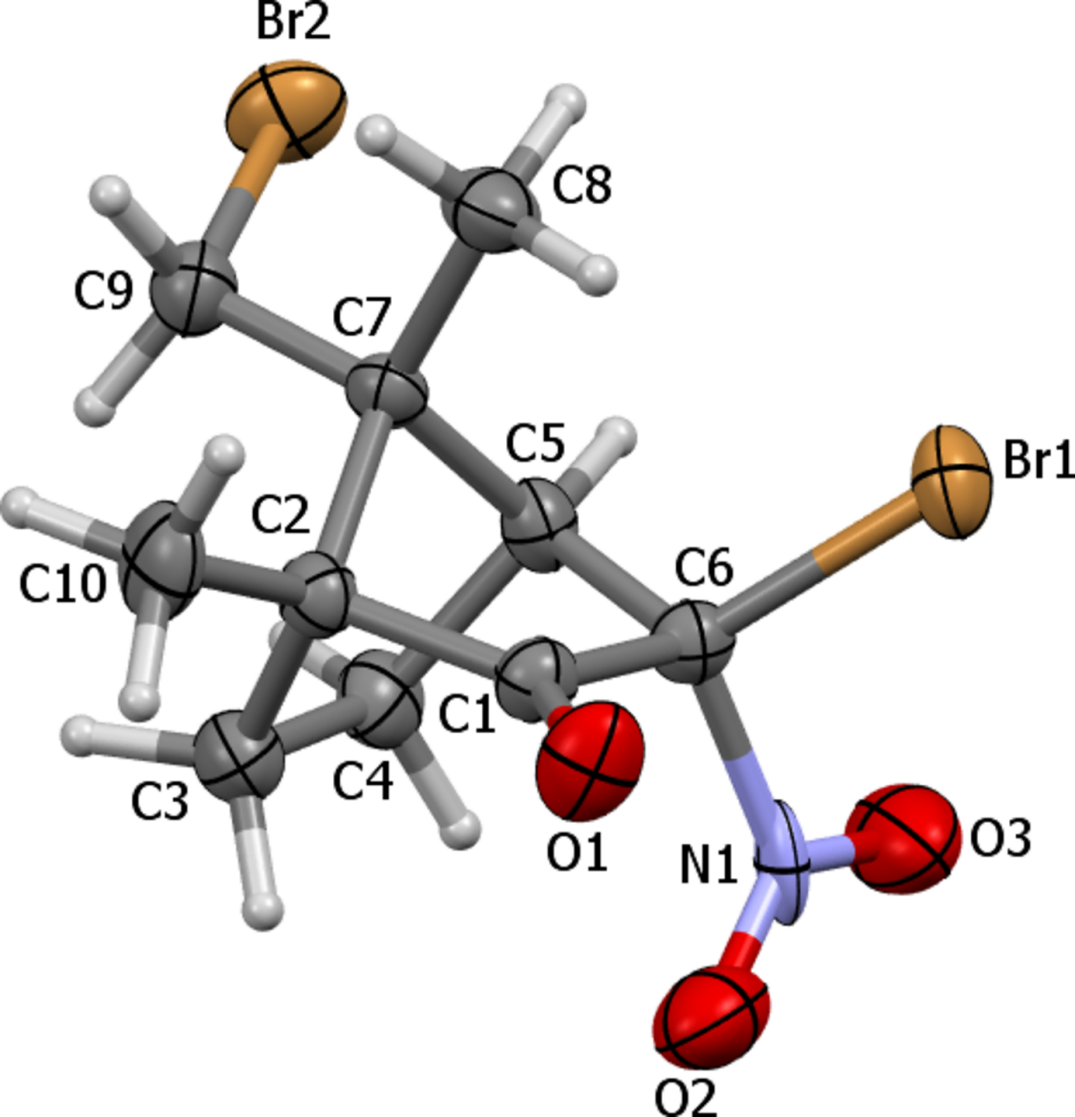
Mol­ecular structure of the title compound with displacement ellipsoids drawn at the 50% probability level.

The title compound (Fig. 1 ▸) crystallizes in the Sohncke space group *P*2_1_2_1_2_1_. The presence of bromine atoms as strong anomalous scatterers in the mol­ecule makes it possible to determine unambiguously the absolute configurations of chiral centers at the C2 (*R*), C5 (*S*), C6 (*R*) and C7 (*R*) atoms by using laboratory Mo *K*α radiation (Flack parameter 0.004 (17) using 680 quotients [(*I*^+^) − (*I*^−^)]/[(*I*^+^) + (*I*^−^)] (Parsons *et al.*, 2013 ▸). The six-membered ring of the bi­cyclo­[2.2.1]-heptan-2-one moiety adopts a boat conformation with puckering parameters *Q* = 0.983 (8), *Θ* = 90.2 (5)°, *Ψ* = 244.0 (4)° (Cremer & Pople, 1975 ▸), whereby the C3, C4, C6 and C1 atoms of this ring deviate from their least-squares plane by 0.0342 Å, while the C2 and C5 atoms deviate from this plane by −0.565 (6) and −0.567 (6) Å, respectively. Both five-membered rings adopt an envelope conformation. In the C2–C3–C4–C5–C7 ring [puckering parameters *Q* = 0.586 (8), *Ψ* = 139.5 (8)°], the C2, C3, C4, C5 atoms deviate from their least-squares plane by 0.0249 Å, while the C7 atom deviates from this plane by 0.377 (5) Å. In the C2–C1–C6–C5–C7 ring [puckering parameters *Q* = 0.588 (8), *Ψ* = 70.7 (7)°], the C2, C1, C6, C5 atoms deviate from their least-squares plane by 0.0116 Å, while the C7 atom deviates from this plane by 0.377 (5) Å. The bromine substituent at the C6 atom has an *exo*-orientation in relation to the bi­cyclo­[2.2.1]-heptan-2-one fragment [the C4—C5—C6—Br1 torsion angle is 165.9 (5)°] The nitro group has an *endo*-orientation and is turned relatively to the C5—C6 bond [the C4—C5—C6—N1 and C5—C6—N1—O3 torsion angles are 47.4 (7)° and 67.4 (8)°, respectively]. The Br2 atom is located in a *syn-clinal* position in relation to the C5—C7 bond [the C5—C7—C9—Br2 torsion angle is −53.9 (7)°].

## 3. Supra­molecular features

**Figure 2:**
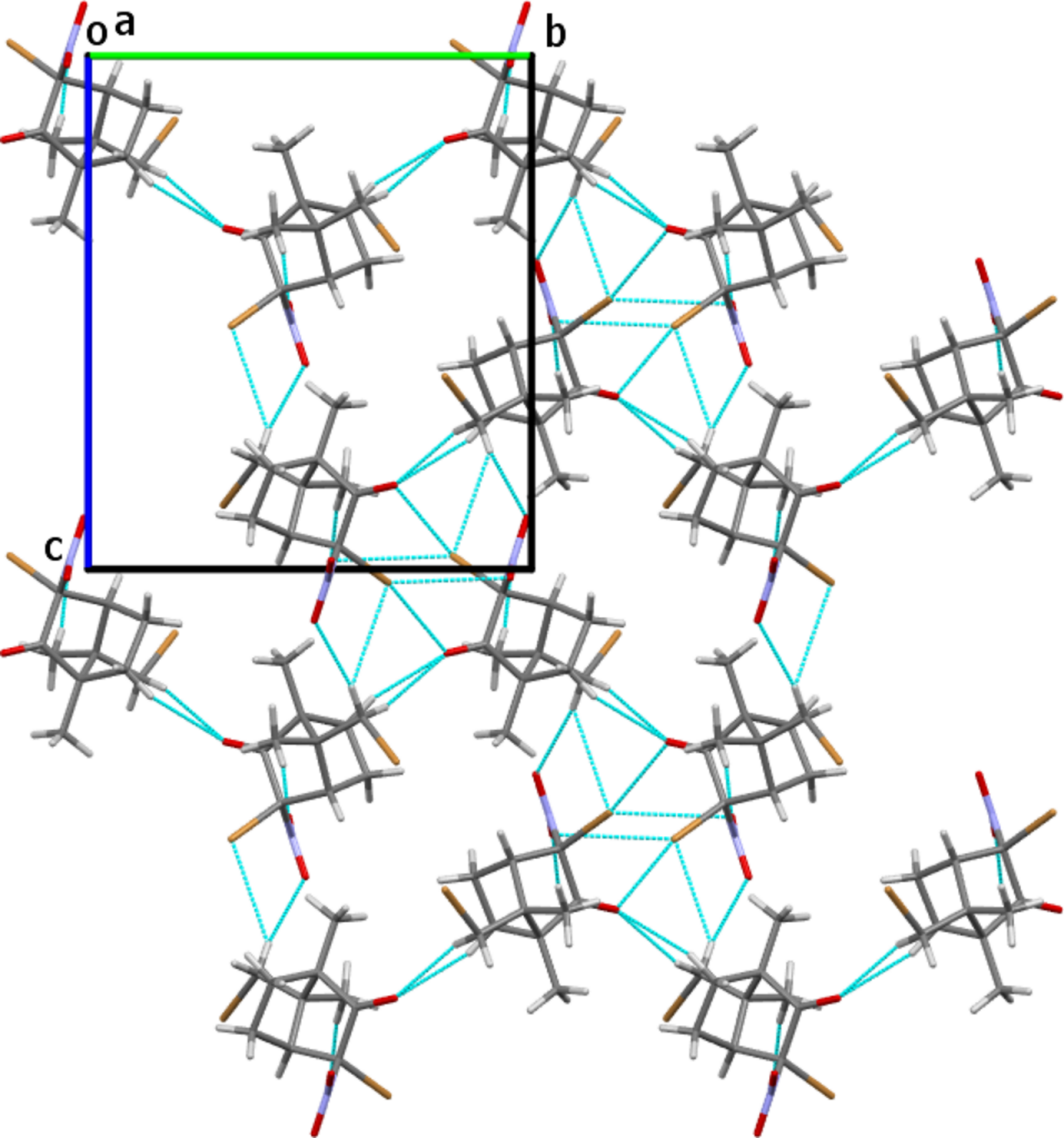
Mol­ecular packing in the crystal structure of the title compound in a projection along the *a* axis. Weak inter­molecular inter­actions are shown as blue dashed lines.

The title compound does not contain any functional groups acting as a strong donor for inter­molecular hydrogen-bonding. However, weak C*sp*^3^—H⋯O and *C*sp^3^—H⋯Br hydrogen bonds are observed in the crystal structure (Table 1 ▸). In addition, the distance between the Br1 and O1 atoms of 3.086 (5) Å (symmetry code −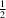 + *x*, 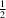 − *y*, 1 − *z*) proved to be shorter than the Br⋯O van der Waals radii sum of 3.37 Å (Hu *et al.*, 2014 ▸). The mutual orientation of the corresponding functional groups of the neighboring mol­ecules [the C6—Br1⋯O1 angle is 167.0 (2)°] allows us to consider this inter­action as a halogen bond (Cavallo *et al.*, 2016 ▸). The weak character of the inter­molecular inter­actions in the extended structure makes it impossible to identify a characteristic structural motif (Fig. 2 ▸).

## 4. Database survey

A search of the Cambridge Structure Database (CSD, version 6.00, last update April 2025; Groom *et al.*, 2016 ▸) revealed only eight structures of bi­cyclo­[2.2.1]heptan derivatives containing geminal bromine and nitro substituents: AWIGIX, AWIGOD, AWIGUJ, AWIHAQ, BRHPCN01 (Lemmerer & Michael, 2011 ▸), BNFNCH (Rerat, 1968 ▸), BRHPCN (Blom *et al.*, 1980 ▸), and BRONCP (Brueckner *et al.*, 1962 ▸). The conformation of the bi­cyclo­[2.2.1]heptane fragment is identical in the structure of the title compound and in all these mol­ecules, despite the fact that none of the similar compounds found in the CSD contain a carbonyl group and, consequently, a carbon atom with *sp*^2^ hybridization in the bicyclic core. The orientation of the geminal bromine and nitro substituents was also found to be identical in the structure of the title compound and previously studied compounds.

## 5. Synthesis and crystallization

A stirred mixture of (1*R*,3*S*,4*S*,7*R*)-3-bromo-7-(bromo­meth­yl)-1,7-dimethyl­[2.2.1]heptan-2-one (98%, Aldrich) (0.76 g, 2.45 mmol) and 60% nitric acid (10 ml) was refluxed under an argon atmosphere for 72 h. Nitric acid was evaporated under reduced pressure and distilled water (50 ml) was added to the residue. The remaining mixture was extracted with toluene (3 × 20 ml). The organic phase was dried over Na_2_SO_4_ and the solvent evaporated under reduced pressure. The crude product was crystallized from 2-propanol; yield 0.55 g (63%) as a white solid. X-ray-quality single crystals of suitable dimensions were obtained by further recrystallization from 2-propanol over a period of 24 h. M.p.: 371–372 K; [α]20/D = +43.8 (*c* 0.5, MeOH).

The relative configuration at the bridge carbon atom connecting the CH_2_Br and CH_3_ groups of the compound was confirmed by comprehensive analysis of the 1D ^1^H-NMR and ^13^C-NMR spectra and results of 2D experiments – COSY, HMBC, HSQC, and NOESY, which were run in CD_3_OD (see supporting information).

## 6. Refinement

Crystal data, data collection and structure refinement details are summarized in Table 2 ▸. Hydrogen atoms were placed at calculated positions and refined as riding with *U*_iso_(H) = *nU*_eq_(C), where *n* = 1.5 for methyl groups and *n* = 1.2 for other H atoms.

## Supplementary Material

Crystal structure: contains datablock(s) I. DOI: 10.1107/S2056989026003592/wm5793sup1.cif

Structure factors: contains datablock(s) I. DOI: 10.1107/S2056989026003592/wm5793Isup2.hkl

Supporting information file. DOI: 10.1107/S2056989026003592/wm5793sup4.pdf

Supporting information file. DOI: 10.1107/S2056989026003592/wm5793Isup4.cml

CCDC reference: 2544283

Additional supporting information:  crystallographic information; 3D view; checkCIF report

## Figures and Tables

**Table 1:** Details of weak inter­molecular hydrogen bonds (Å, °)

Inter­action	Symmetry operation	H⋯*A^*a*^*	*D*⋯*A*	*D*—H⋯*A*
C8—H8*B*⋯O2	−1 + *x*, *y*, *z*	2.49	3.46 (1)	169
C9—H9*B*⋯O1	1 − *x*, 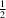 + *y*, 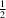 − *z*	2.44	3.364 (9)	154
C9—H9*A*⋯Br1	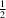 − *x*, 1 − *y*, − 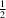 + *z*	3.04	3.730 (7)	128

Note: (*a* The van der Waals radii sums are: H⋯O 2.58 Å, H⋯Br 3.07 Å (Hu *et al.*, 2014 ▸).

**Table 2:** Experimental details

Crystal data	
Chemical formula	C_10_H_13_Br_2_NO_3_
*M*_r_	355.03
Crystal system, space group	Orthorhombic, *P*2_1_2_1_2_1_
Temperature (K)	173
*a*, *b*, *c* (Å)	7.5173 (11), 12.0316 (18), 13.9292 (19)
*V* (Å^3^)	1259.8 (3)
*Z*	4
Radiation type	Mo *K*α
μ (mm^−1^)	6.43
Crystal size (mm)	0.24 × 0.14 × 0.11

Data collection	
Diffractometer	Bruker APEXII CCD
Absorption correction	Multi-scan (*SADABS*; Krause *et al.*, 2015 ▸)
*T*_min_, *T*_max_	0.343, 0.746
No. of measured, independent and observed [*I* > 2σ(*I*)] reflections	10299, 2890, 2052
*R*_int_	0.077
(sin θ/λ)_max_ (Å^−1^)	0.650

Refinement	
*R*[*F*^2^ > 2σ(*F*^2^)], *wR*(*F*^2^), *S*	0.040, 0.089, 0.97
No. of reflections	2890
No. of parameters	147
H-atom treatment	H-atom parameters constrained
Δρ_max_, Δρ_min_ (e Å^−3^)	0.62, −0.64
Absolute structure	Flack *x* determined using 680 quotients [(*I*^+^)−(*I*^−^)]/[(*I*^+^)+(*I*^−^)] (Parsons *et al.*, 2013 ▸)
Absolute structure parameter	0.004 (17)

Computer programs: *SAINT* and *APEX4* (Bruker, 2021 ▸), *SHELXT* (Sheldrick, 2015*a* ▸), *SHELXL* (Sheldrick, 2015*b* ▸), *OLEX2* (Dolomanov *et al.*, 2009 ▸), *Mercury* (Macrae *et al.*, 2020 ▸) and *publCIF* (Westrip, 2010 ▸).

## supplementary crystallographic information

### (1R,3R,4S,7R)-3-Bromo-7-(bromomethyl)-1,7-dimethyl-3-nitrobicyclo[2.2.1]heptan-2-one Crystal data

**Table:**

C_10_H_13_Br_2_NO_3_	*D*_x_ = 1.872 Mg m^−^^3^
*M**_r_* = 355.03	Mo *K*α radiation, λ = 0.71073 Å
Orthorhombic, *P*2_1_2_1_2_1_	Cell parameters from 1737 reflections
*a* = 7.5173 (11) Å	θ = 2.2–21.0°
*b* = 12.0316 (18) Å	µ = 6.43 mm^−^^1^
*c* = 13.9292 (19) Å	*T* = 173 K
*V* = 1259.8 (3) Å^3^	Prism, colourless
*Z* = 4	0.24 × 0.14 × 0.11 mm
*F*(000) = 696	

### (1R,3R,4S,7R)-3-Bromo-7-(bromomethyl)-1,7-dimethyl-3-nitrobicyclo[2.2.1]heptan-2-one Data collection

**Table:**

Bruker APEXII CCD diffractometer	2052 reflections with *I* > 2σ(*I*)
φ and ω scans	*R*_int_ = 0.077
Absorption correction: multi-scan (*SADABS*; Krause *et al.*, 2015)	θ_max_ = 27.5°, θ_min_ = 2.2°
*T*_min_ = 0.343, *T*_max_ = 0.746	*h* = −9→9
10299 measured reflections	*k* = −15→13
2890 independent reflections	*l* = −18→18

### (1R,3R,4S,7R)-3-Bromo-7-(bromomethyl)-1,7-dimethyl-3-nitrobicyclo[2.2.1]heptan-2-one Refinement

**Table:**

Refinement on *F*^2^	Hydrogen site location: inferred from neighbouring sites
Least-squares matrix: full	H-atom parameters constrained
*R*[*F*^2^ > 2σ(*F*^2^)] = 0.040	*w* = 1/[σ^2^(*F*_o_^2^)] where *P* = (*F*_o_^2^ + 2*F*_c_^2^)/3
*wR*(*F*^2^) = 0.089	(Δ/σ)_max_ < 0.001
*S* = 0.97	Δρ_max_ = 0.62 e Å^−^^3^
2890 reflections	Δρ_min_ = −0.64 e Å^−^^3^
147 parameters	Absolute structure: Flack *x* determined using 680 quotients [(*I*^+^)-(*I*^-^)]/[(*I*^+^)+(*I*^-^)] (Parsons *et al.* (2013)
0 restraints	Absolute structure parameter: 0.004 (17)
Primary atom site location: dual	

### (1R,3R,4S,7R)-3-Bromo-7-(bromomethyl)-1,7-dimethyl-3-nitrobicyclo[2.2.1]heptan-2-one Special details

**Table:**

Geometry. All esds (except the esd in the dihedral angle between two l.s. planes) are estimated using the full covariance matrix. The cell esds are taken into account individually in the estimation of esds in distances, angles and torsion angles; correlations between esds in cell parameters are only used when they are defined by crystal symmetry. An approximate (isotropic) treatment of cell esds is used for estimating esds involving l.s. planes.

### (1R,3R,4S,7R)-3-Bromo-7-(bromomethyl)-1,7-dimethyl-3-nitrobicyclo[2.2.1]heptan-2-one Fractional atomic coordinates and isotropic or equivalent isotropic displacement parameters (Å^2^)

**Table:**

	*x*	*y*	*z*	*U*_iso_*/*U*_eq_	
Br1	0.32898 (11)	0.32332 (6)	0.52580 (5)	0.0388 (2)	
Br2	0.01737 (10)	0.69501 (7)	0.37509 (6)	0.0470 (3)	
C1	0.5113 (9)	0.3955 (6)	0.3535 (5)	0.0273 (16)	
C2	0.4454 (9)	0.4818 (6)	0.2852 (5)	0.0272 (17)	
C3	0.5662 (10)	0.5843 (6)	0.3040 (5)	0.0359 (19)	
H3A	0.692829	0.562138	0.307839	0.043*	
H3B	0.552230	0.640438	0.252561	0.043*	
C4	0.4999 (10)	0.6299 (6)	0.4014 (5)	0.0342 (18)	
H4A	0.598378	0.634616	0.448351	0.041*	
H4B	0.445991	0.704500	0.393679	0.041*	
C5	0.3596 (10)	0.5440 (6)	0.4332 (5)	0.0283 (16)	
H5	0.277415	0.569717	0.485126	0.034*	
C6	0.4610 (9)	0.4356 (6)	0.4554 (5)	0.0279 (17)	
C7	0.2676 (9)	0.5160 (6)	0.3356 (5)	0.0257 (16)	
C8	0.1277 (9)	0.4220 (7)	0.3342 (6)	0.037 (2)	
H8A	0.077222	0.415592	0.269592	0.056*	
H8B	0.032806	0.439198	0.380088	0.056*	
H8C	0.184449	0.351663	0.352065	0.056*	
C9	0.1825 (11)	0.6178 (6)	0.2886 (5)	0.0345 (17)	
H9A	0.117560	0.594186	0.230288	0.041*	
H9B	0.277332	0.670043	0.268659	0.041*	
C10	0.4378 (11)	0.4419 (7)	0.1817 (5)	0.044 (2)	
H10A	0.557036	0.419484	0.160908	0.065*	
H10B	0.394633	0.502195	0.140564	0.065*	
H10C	0.356832	0.378299	0.177047	0.065*	
N1	0.6294 (10)	0.4597 (5)	0.5205 (4)	0.0320 (15)	
O1	0.5882 (7)	0.3095 (5)	0.3363 (3)	0.0434 (13)	
O2	0.7630 (8)	0.4518 (5)	0.4830 (5)	0.0531 (16)	
O3	0.5953 (8)	0.4899 (6)	0.5995 (4)	0.0600 (18)	

### (1R,3R,4S,7R)-3-Bromo-7-(bromomethyl)-1,7-dimethyl-3-nitrobicyclo[2.2.1]heptan-2-one Atomic displacement parameters (Å^2^)

**Table:**

	*U*^11^	*U*^22^	*U*^33^	*U*^12^	*U*^13^	*U*^23^
Br1	0.0510 (5)	0.0335 (4)	0.0318 (4)	−0.0061 (4)	0.0032 (4)	0.0069 (4)
Br2	0.0417 (5)	0.0446 (5)	0.0546 (5)	0.0128 (4)	−0.0004 (4)	−0.0050 (4)
C1	0.028 (4)	0.025 (4)	0.029 (4)	0.003 (3)	0.000 (3)	0.000 (3)
C2	0.029 (4)	0.028 (4)	0.024 (4)	−0.001 (3)	0.001 (3)	−0.001 (3)
C3	0.035 (4)	0.038 (5)	0.035 (5)	−0.003 (3)	0.001 (3)	0.004 (4)
C4	0.040 (4)	0.024 (4)	0.039 (4)	−0.005 (4)	−0.003 (4)	0.004 (3)
C5	0.034 (4)	0.026 (4)	0.026 (4)	0.001 (3)	0.006 (3)	0.002 (3)
C6	0.028 (4)	0.025 (4)	0.030 (4)	0.002 (3)	0.000 (3)	−0.001 (3)
C7	0.023 (4)	0.028 (4)	0.026 (4)	0.002 (3)	0.003 (3)	0.001 (3)
C8	0.029 (4)	0.042 (5)	0.040 (5)	−0.006 (4)	0.003 (4)	0.000 (4)
C9	0.037 (4)	0.036 (4)	0.031 (4)	0.001 (4)	−0.001 (4)	0.004 (3)
C10	0.056 (5)	0.054 (5)	0.021 (4)	0.009 (4)	0.004 (4)	0.000 (4)
N1	0.058 (5)	0.025 (3)	0.014 (3)	0.007 (3)	0.014 (3)	0.005 (3)
O1	0.056 (3)	0.035 (3)	0.039 (3)	0.016 (3)	0.000 (3)	−0.007 (3)
O2	0.046 (4)	0.053 (4)	0.061 (4)	−0.001 (3)	−0.016 (3)	0.005 (4)
O3	0.051 (4)	0.086 (5)	0.043 (4)	−0.012 (3)	−0.007 (3)	0.000 (4)

### (1R,3R,4S,7R)-3-Bromo-7-(bromomethyl)-1,7-dimethyl-3-nitrobicyclo[2.2.1]heptan-2-one Geometric parameters (Å, º)

**Table:**

Br1—C6	1.941 (7)	C5—C6	1.542 (9)
Br2—C9	1.963 (7)	C5—C7	1.562 (9)
C1—C2	1.493 (10)	C6—N1	1.584 (9)
C1—C6	1.546 (10)	C7—C8	1.544 (10)
C1—O1	1.209 (8)	C7—C9	1.529 (10)
C2—C3	1.553 (10)	C8—H8A	0.9800
C2—C7	1.565 (9)	C8—H8B	0.9800
C2—C10	1.520 (10)	C8—H8C	0.9800
C3—H3A	0.9900	C9—H9A	0.9900
C3—H3B	0.9900	C9—H9B	0.9900
C3—C4	1.546 (10)	C10—H10A	0.9800
C4—H4A	0.9900	C10—H10B	0.9800
C4—H4B	0.9900	C10—H10C	0.9800
C4—C5	1.543 (10)	N1—O2	1.136 (7)
C5—H5	1.0000	N1—O3	1.187 (8)
			
C2—C1—C6	106.7 (6)	C5—C6—C1	101.6 (5)
O1—C1—C2	128.8 (6)	C5—C6—N1	110.8 (5)
O1—C1—C6	124.5 (6)	N1—C6—Br1	104.3 (4)
C1—C2—C3	104.5 (6)	C5—C7—C2	94.0 (5)
C1—C2—C7	100.4 (5)	C8—C7—C2	112.5 (6)
C1—C2—C10	113.4 (6)	C8—C7—C5	118.0 (6)
C3—C2—C7	102.4 (6)	C9—C7—C2	112.1 (6)
C10—C2—C3	115.6 (6)	C9—C7—C5	112.7 (6)
C10—C2—C7	118.5 (6)	C9—C7—C8	107.2 (6)
C2—C3—H3A	111.0	C7—C8—H8A	109.5
C2—C3—H3B	111.0	C7—C8—H8B	109.5
H3A—C3—H3B	109.0	C7—C8—H8C	109.5
C4—C3—C2	104.0 (6)	H8A—C8—H8B	109.5
C4—C3—H3A	111.0	H8A—C8—H8C	109.5
C4—C3—H3B	111.0	H8B—C8—H8C	109.5
C3—C4—H4A	111.0	Br2—C9—H9A	109.1
C3—C4—H4B	111.0	Br2—C9—H9B	109.1
H4A—C4—H4B	109.0	C7—C9—Br2	112.4 (5)
C5—C4—C3	103.6 (6)	C7—C9—H9A	109.1
C5—C4—H4A	111.0	C7—C9—H9B	109.1
C5—C4—H4B	111.0	H9A—C9—H9B	107.9
C4—C5—H5	115.0	C2—C10—H10A	109.5
C4—C5—C7	101.3 (5)	C2—C10—H10B	109.5
C6—C5—C4	106.7 (6)	C2—C10—H10C	109.5
C6—C5—H5	115.0	H10A—C10—H10B	109.5
C6—C5—C7	102.2 (5)	H10A—C10—H10C	109.5
C7—C5—H5	115.0	H10B—C10—H10C	109.5
C1—C6—Br1	111.8 (5)	O2—N1—C6	115.3 (6)
C1—C6—N1	112.8 (5)	O2—N1—O3	130.0 (8)
C5—C6—Br1	115.9 (5)	O3—N1—C6	114.5 (6)
			
Br1—C6—N1—O2	126.5 (6)	C5—C6—N1—O2	−108.1 (7)
Br1—C6—N1—O3	−58.0 (7)	C5—C6—N1—O3	67.4 (8)
C1—C2—C3—C4	−73.6 (7)	C5—C7—C9—Br2	−53.9 (7)
C1—C2—C7—C5	54.9 (6)	C6—C1—C2—C3	69.1 (7)
C1—C2—C7—C8	−67.7 (7)	C6—C1—C2—C7	−36.8 (7)
C1—C2—C7—C9	171.3 (6)	C6—C1—C2—C10	−164.1 (6)
C1—C6—N1—O2	4.9 (9)	C6—C5—C7—C2	−54.3 (6)
C1—C6—N1—O3	−179.5 (6)	C6—C5—C7—C8	63.9 (8)
C2—C1—C6—Br1	126.5 (5)	C6—C5—C7—C9	−170.2 (6)
C2—C1—C6—C5	2.3 (7)	C7—C2—C3—C4	30.7 (7)
C2—C1—C6—N1	−116.3 (6)	C7—C5—C6—Br1	−88.1 (5)
C2—C3—C4—C5	4.8 (7)	C7—C5—C6—C1	33.3 (6)
C2—C7—C9—Br2	−158.4 (5)	C7—C5—C6—N1	153.3 (5)
C3—C2—C7—C5	−52.6 (6)	C8—C7—C9—Br2	77.6 (6)
C3—C2—C7—C8	−175.3 (6)	C10—C2—C3—C4	161.0 (6)
C3—C2—C7—C9	63.8 (7)	C10—C2—C7—C5	178.8 (6)
C3—C4—C5—C6	67.8 (7)	C10—C2—C7—C8	56.2 (9)
C3—C4—C5—C7	−38.7 (7)	C10—C2—C7—C9	−64.8 (8)
C4—C5—C6—Br1	165.9 (5)	O1—C1—C2—C3	−111.5 (8)
C4—C5—C6—C1	−72.6 (6)	O1—C1—C2—C7	142.7 (7)
C4—C5—C6—N1	47.4 (7)	O1—C1—C2—C10	15.3 (11)
C4—C5—C7—C2	55.8 (6)	O1—C1—C6—Br1	−52.9 (9)
C4—C5—C7—C8	173.9 (6)	O1—C1—C6—C5	−177.2 (7)
C4—C5—C7—C9	−60.2 (7)	O1—C1—C6—N1	64.2 (9)
