# Supplementary material for: Mol­ecular and crystal structure of (1R,3R,4S,7R)-3-bromo-7-(bromo­meth­yl)-1,7-dimethyl-3-nitro­bi­cyclo­[2.2.1]heptan-2-one
Source: Acta Crystallogr E Crystallogr Commun. 2026 Apr 14;82(Pt 5):473–6. doi: 10.1107/S2056989026003592 (PMC13148212; doi:10.1107/S2056989026003592)
Supplement: Supplementary file 3 [file e-82-00473-sup4.pdf]

Relative configuration at the bridgehead carbon atom connecting the CH_2Br and CH_3 groups of the compound was confirmed by comprehensive analysis of the 1D ^{1}H-NMR and ^{13}C-NMR spectra and results of 2D experiments – COSY, HMBC, HSQC, and NOESY, which were run in CD_3OD. In this solvent, most of the non-equivalent proton and carbon signals are non-overlapping. First, absolute NMR signal assignments were done. ^{1}H NMR signals of the adjacent 7-CH_2Br and 7-CH_3 groups (IUPAC atom numeration), important for interpretation of the NOESY experiment, were assigned by their chemical shift values, splitting patterns and clear correlation of one of the CH_2Br proton signals with the CH_3 group signal in the COSY spectrum (the doublet at 3.81 ppm correlates with the singlet at 1.34 ppm). Assignments of the ^{1}H NMR signals of the methyl groups were also confirmed by HMBC (proton singlet at 1.07 ppm correlates with carbon signals at 198.5, 27.3, and 47.5 ppm, that indicates that the proton signal stems from 1-CH_3). In the NOESY spectrum, strong correlations between a 7-CH_2Br proton signal and two signals of 5-CH_2 and 6-CH_2 protons are observed (3.81 ppm to 2.12 ppm and 1.94 ppm correlations). These NOE correlations unambiguously prove that CH_2Br and CH_2-CH_2 fragments are in close vicinity to each other. NMR signal assignments are the following (IUPAC numeration) ^{1}H NMR (400 MHz, CD_3OD): δ = 3.81 (d, J = 10.5 Hz, 1H, 7-CHHBr), 3.36 (d, J = 10.5 Hz, 1H, 7-CHHBr), 3.14 (d, J = 4 Hz, 1H, 4-CH), 2.12 (ddd, 1H, 5-CHH), 1.34 (ddd, 1H, 5-CHH), 1.69 (ddd, 1H, 6-CHH), 1.94 (m, 1H, 6-CHH), 1.07 (s, 1-CH3), 1.34 (s, 3H, 7-CH3); ^{13}C NMR (100 MHz, CD_3OD): δ = 198.5 (2-C), 90.7 (3-C), 54.4 (4-CH), 23.5 (5-CH2), 27.3 (6-CH2), 59.2 (1-C), 47.5 (7-C), 19.1 (7-CH3), 38.4 (7-CH2Br).

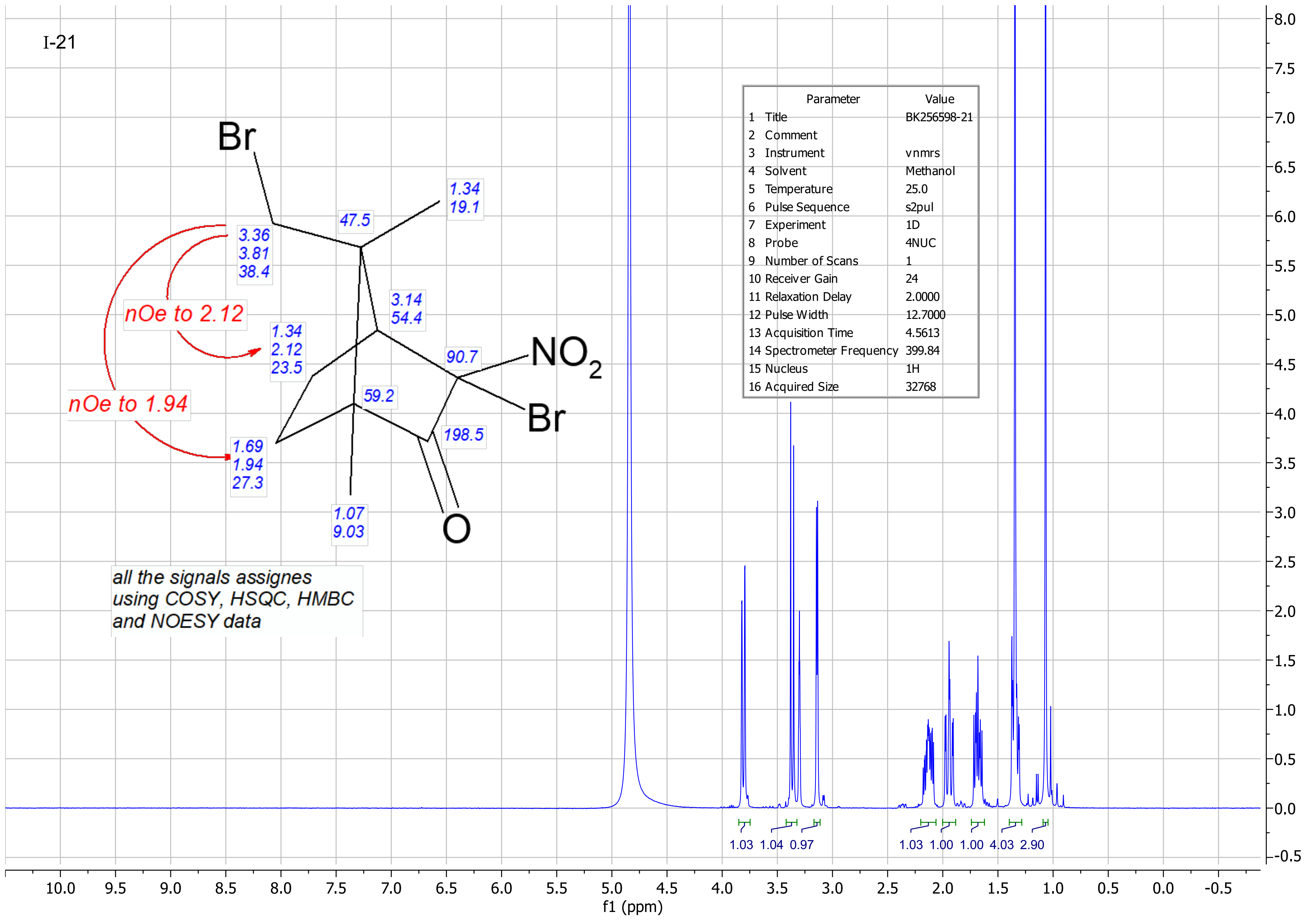

	Parameter	Value
1	Title	BK256598-21
2	Comment	
3	Instrument	vnmrs
4	Solvent	Methanol
5	Temperature	25.0
6	Pulse Sequence	s2pul
7	Experiment	1D
8	Probe	4NUC
9	Number of Scans	1
10	Receiver Gain	24
11	Relaxation Delay	2.0000
12	Pulse Width	12.7000
13	Acquisition Time	4.5613
14	Spectrometer Frequency	399.84
15	Nucleus	1H
16	Acquired Size	32768

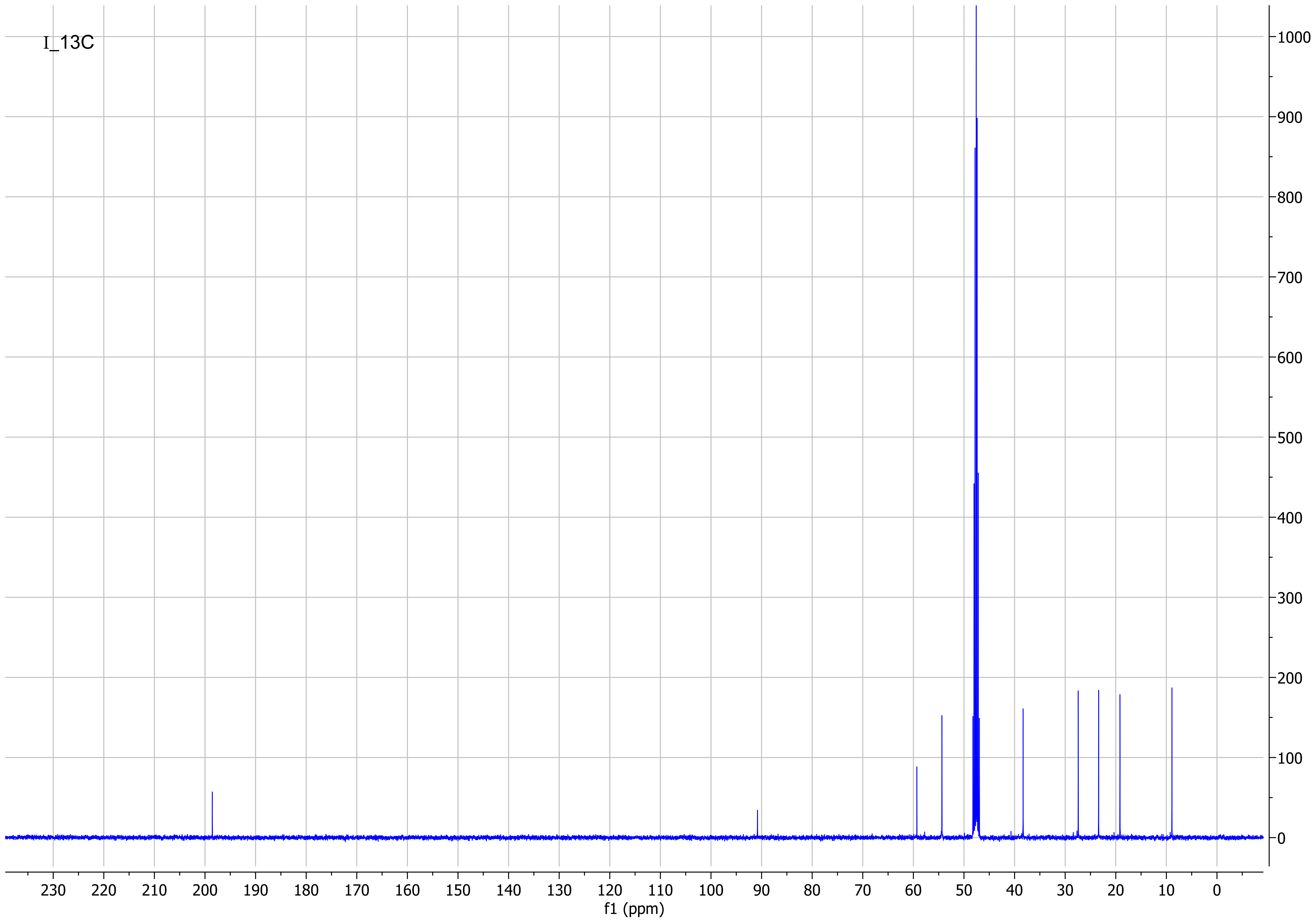
I_13C
f1 (ppm)

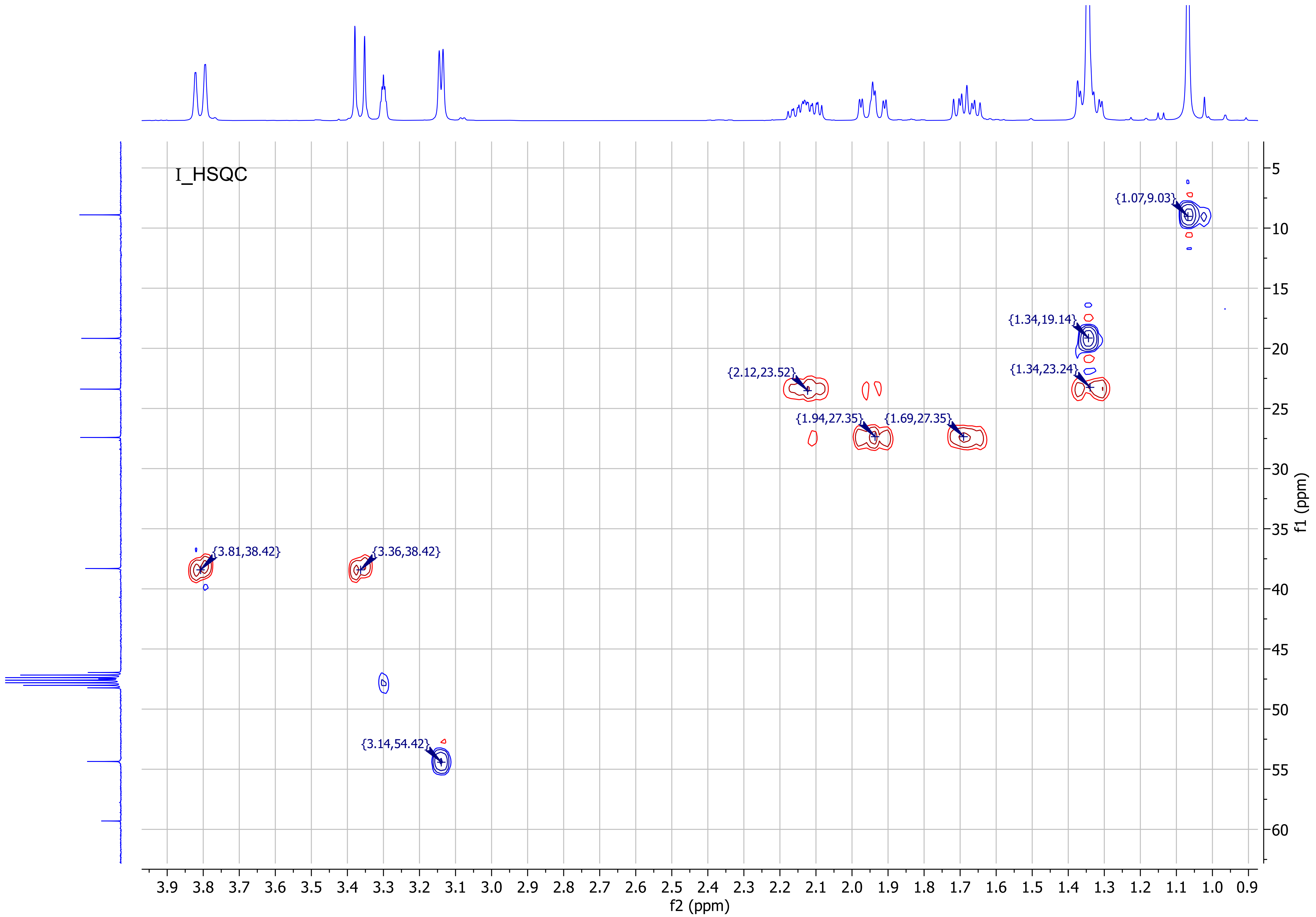

I_HSQC
{1.07,9.03}
{1.34,19.14}
{1.34,23.24}
{2.12,23.52}
{1.94,27.35}
{1.69,27.35}
{3.81,38.42}
{3.36,38.42}
{3.14,54.42}
f2 (ppm)
f1 (ppm)

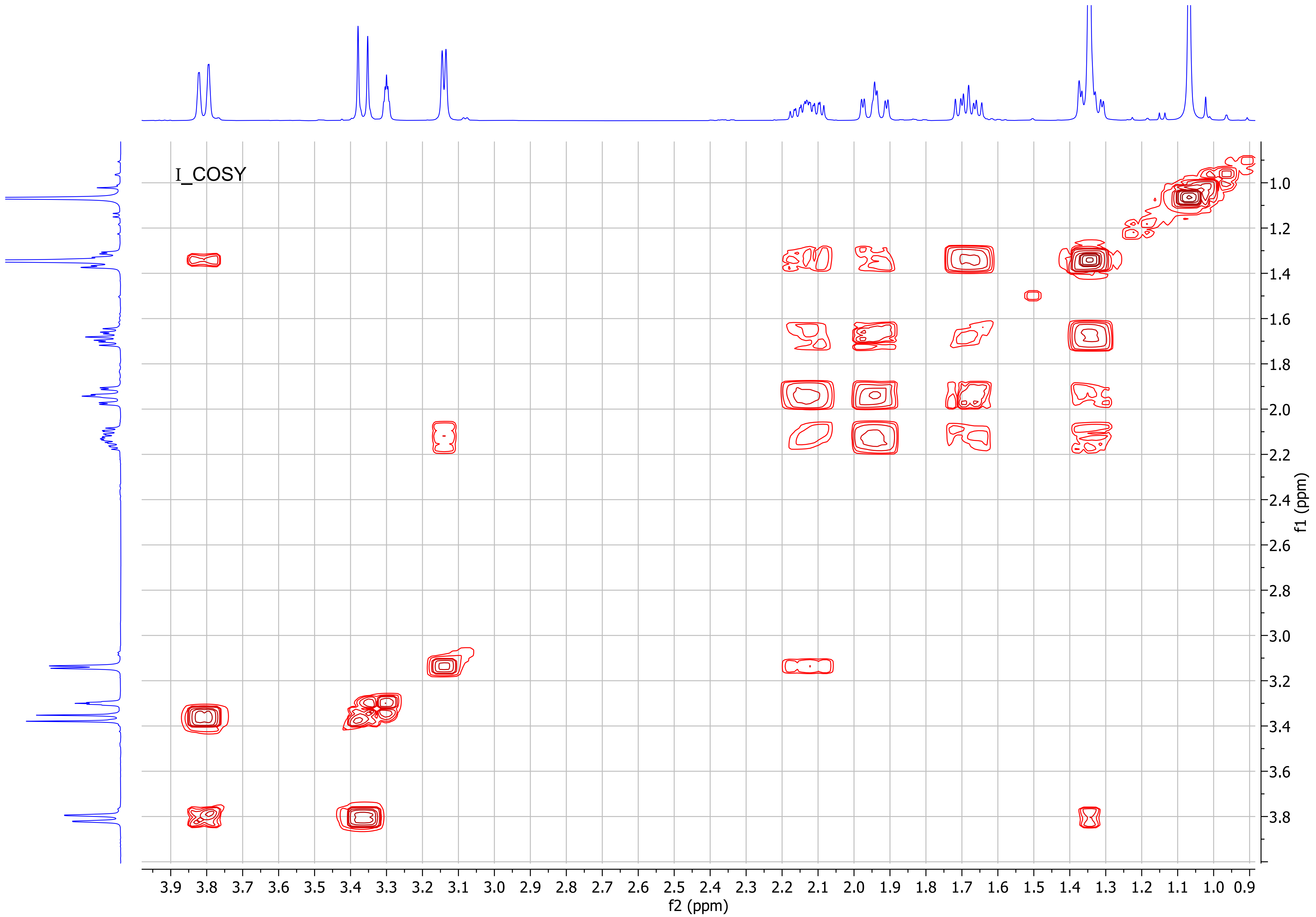

I_COSY
3.9
3.8
3.7
3.6
3.5
3.4
3.3
3.2
3.1
3.0
2.9
2.8
2.7
2.6
2.5
2.4
2.3
2.2
2.1
2.0
1.9
1.8
1.7
1.6
1.5
1.4
1.3
1.2
1.1
1.0
0.9
f2 (ppm)
1.0
1.2
1.4
1.6
1.8
2.0
2.2
2.4
2.6
2.8
3.0
3.2
3.4
3.6
3.8
f1 (ppm)

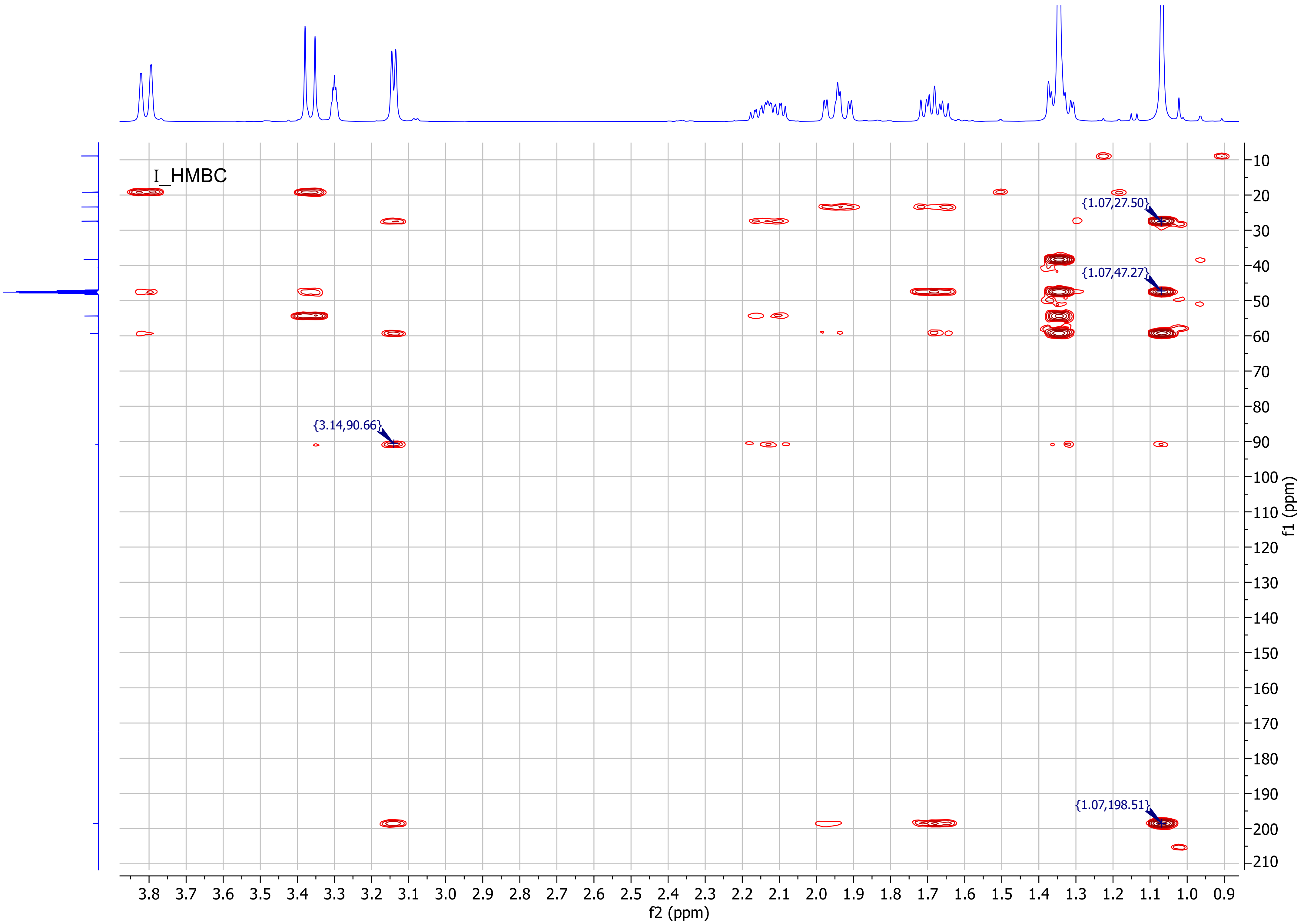

I_HMBC
{1.07,27.50}
{1.07,47.27}
{3.14,90.66}
{1.07,198.51}
f2 (ppm)
f1 (ppm)

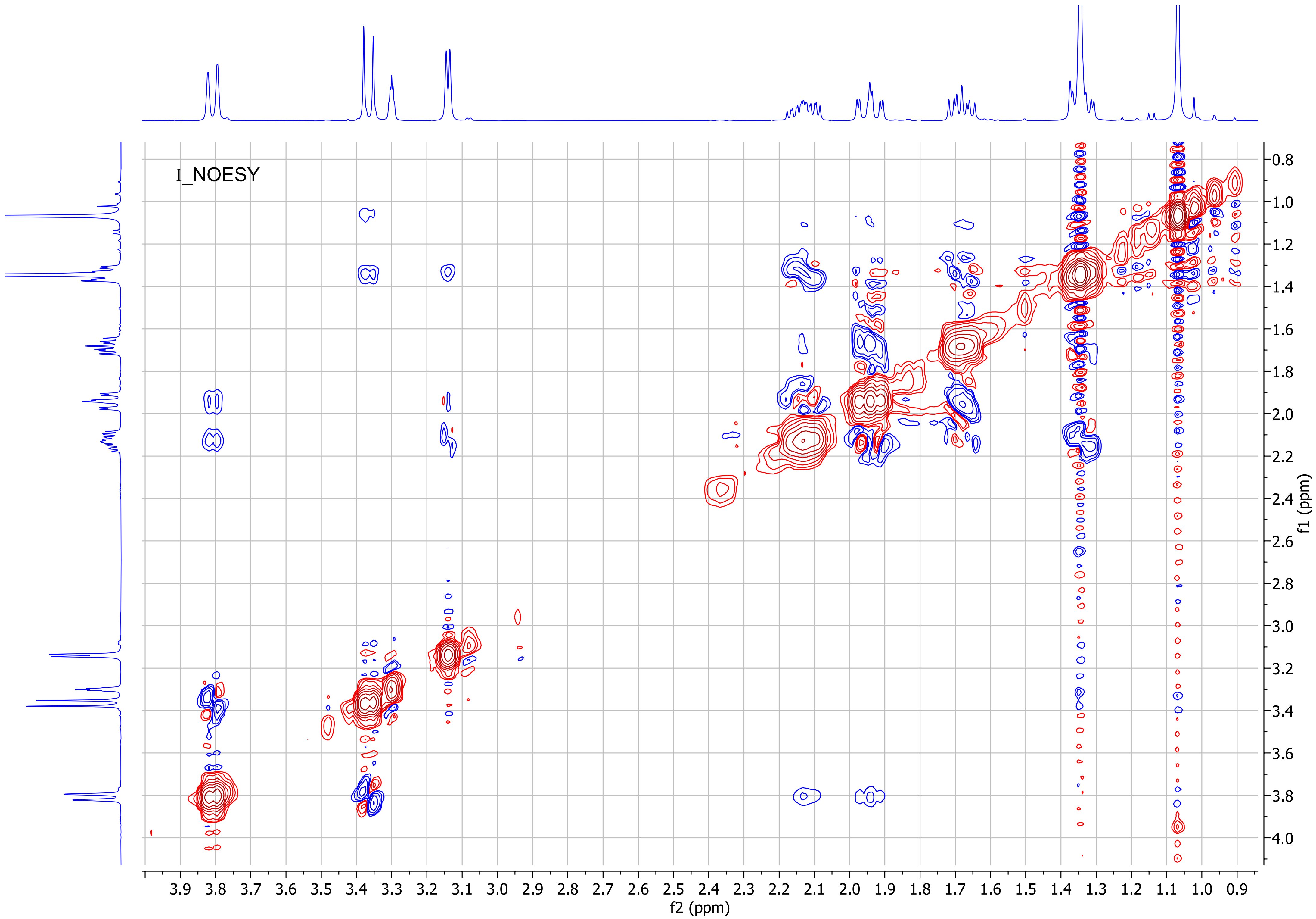
I_NOESY
f2 (ppm)
f1 (ppm)
3.9 3.8 3.7 3.6 3.5 3.4 3.3 3.2 3.1 3.0 2.9 2.8 2.7 2.6 2.5 2.4 2.3 2.2 2.1 2.0 1.9 1.8 1.7 1.6 1.5 1.4 1.3 1.2 1.1 1.0 0.9
0.8 1.0 1.2 1.4 1.6 1.8 2.0 2.2 2.4 2.6 2.8 3.0 3.2 3.4 3.6 3.8 4.0
